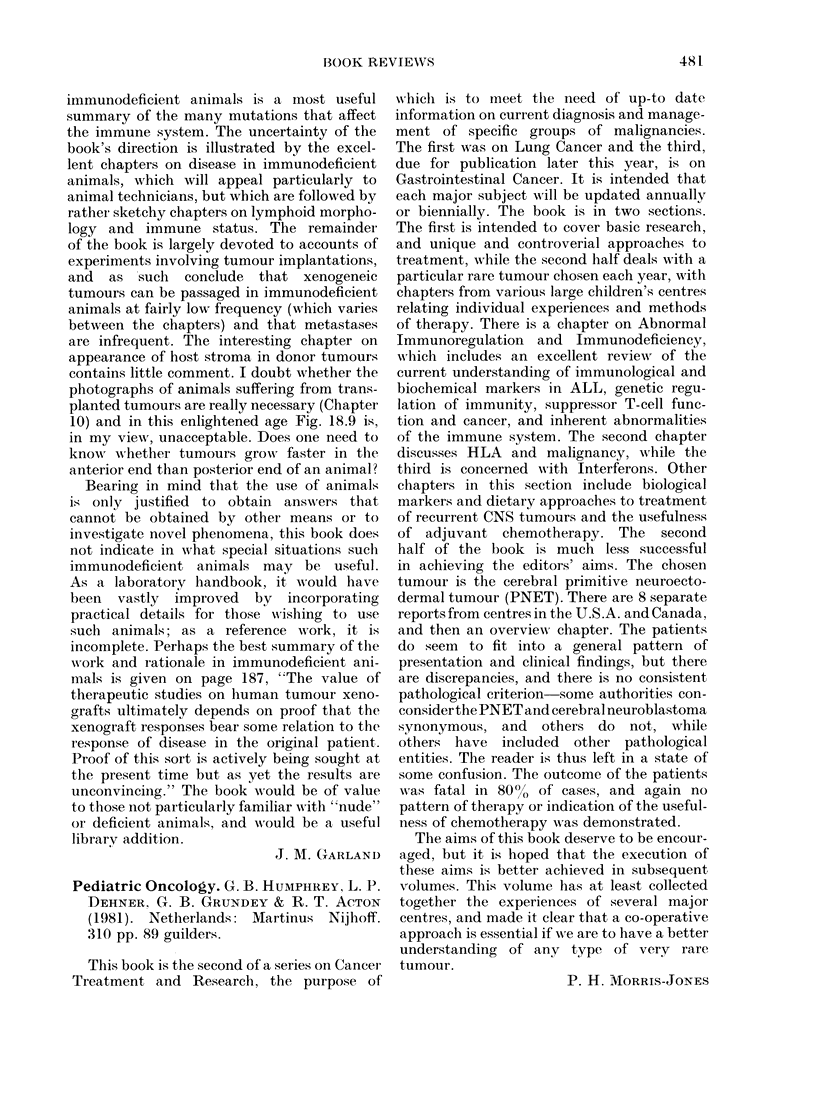# Pediatric Oncology

**Published:** 1981-09

**Authors:** P. H. Morris-Jones


**Pediatric Oncology.** G. B. HUMPHREY, L. P. DEHNER, G. B. GRUNDEY & R. T. ACTON (1981). Netherlands: Martinus Nijhoff. 310 pp. 89 guilders.

This book is the second of a series on Cancer Treatment and Research, the purpose of which is to meet the need of up-to date information on current diagnosis and management of specific groups of malignancies. The first was on Lung Cancer and the third, due for publication later this year, is on Gastrointestinal Cancer. It is intended that each major subject will be updated annually or biennially. The book is in two sections. The first is intended to cover basic research, and unique and controverial approaches to treatment, while the second half deals with a particular rare tumour chosen each year, with chapters from various large children's centres relating individual experiences and methods of therapy. There is a chapter on Abnormal Immunoregulation and Immunodeficiency, which includes an excellent review of the current understanding of immunological and biochemical markers in ALL, genetic regulation of immunity, suppressor T-cell function and cancer, and inherent abnormalities of the immune system. The second chapter discusses HLA and malignancy, while the third is concerned with Interferons. Other chapters in this section include biological markers and dietary approaches to treatment of recurrent CNS tumours and the usefulness of adjuvant chemotherapy. The second half of the book is much less successful in achieving the editors' aims. The chosen tumour is the cerebral primitive neuroectodermal tumour (PNET). There are 8 separate reports from centres in the U.S.A. and Canada, and then an overview chapter. The patients do seem to fit into a general pattern of presentation and clinical findings, but there are discrepancies, and there is no consistent pathological criterion—some authorities conconsider the PNET and cerebral neuroblastoma synonymous, and others do not, while others have included other pathological entities. The reader is thus left in a state of some confusion. The outcome of the patients was fatal in 80% of cases, and again no pattern of therapy or indication of the usefulness of chemotherapy was demonstrated.

The aims of this book deserve to be encouraged, but it is hoped that the execution of these aims is better achieved in subsequent volumes. This volume has at least collected together the experiences of several major centres, and made it clear that a co-operative approach is essential if we are to have a better understanding of any type of very rare tumour.

P. H. MORRIS-JONES